# Supplementary figures and images for: Process evaluation of the HIV+D intervention for integrating the management of depression in routine HIV care in Uganda
Source: PLOS Ment Health. 2024 Jun 4;1(1):e0000009. doi: 10.1371/journal.pmen.0000009 (PMC12798554; doi:10.1371/journal.pmen.0000009)

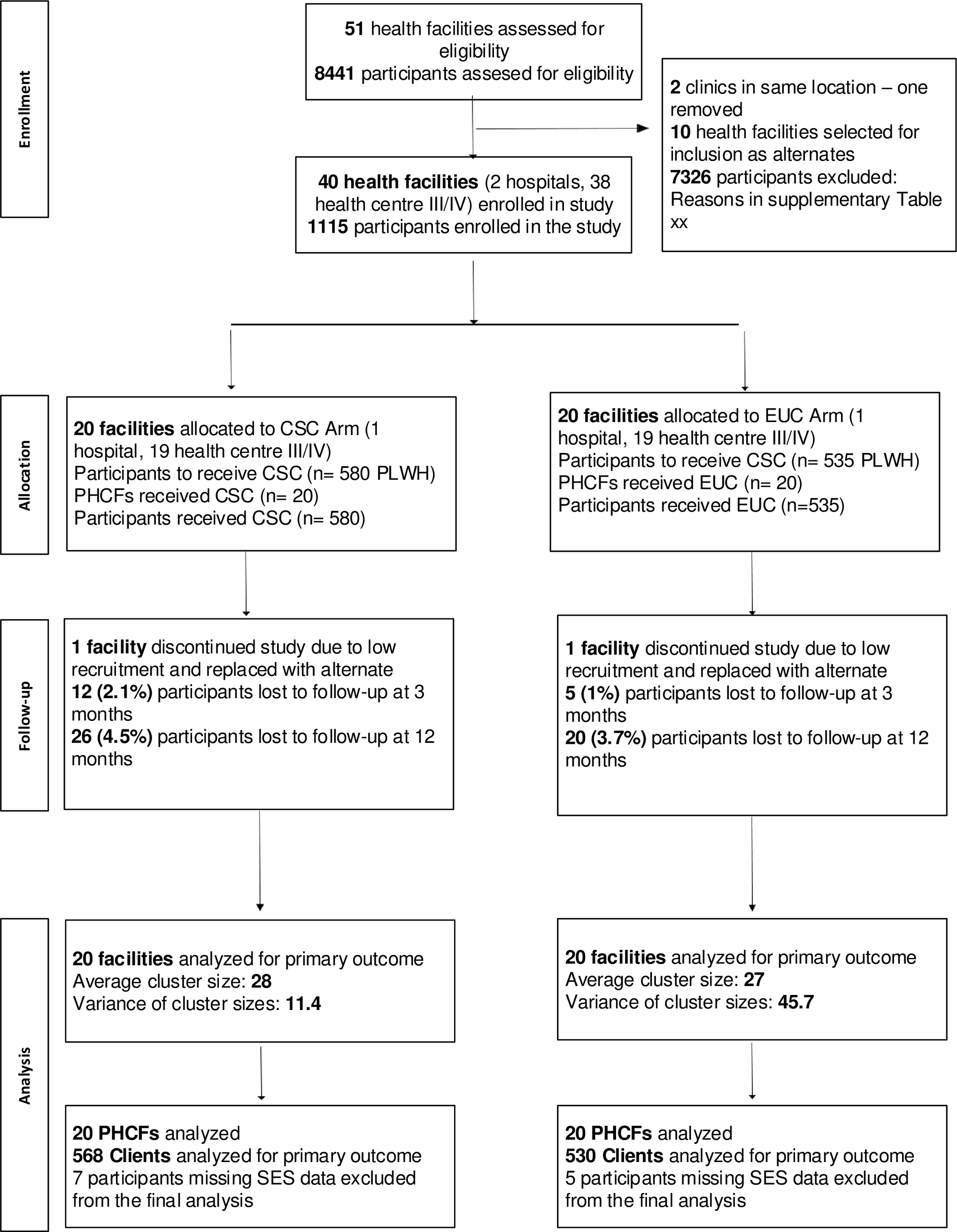

Supplement: S1 Fig — Visual representation of the steps involved (progress and phases) for the HIV+D trial. (TIF) [file pmen.0000009.s002.tif]
