# Supplementary material for: Process evaluation of the HIV+D intervention for integrating the management of depression in routine HIV care in Uganda
Source: PLOS Ment Health. 2024 Jun 4;1(1):e0000009. doi: 10.1371/journal.pmen.0000009 (PMC12798554; doi:10.1371/journal.pmen.0000009)
Supplement: S1 Text — Article about the HIV+D study, highlighting the rationale and objectives of the study, methodology, data management and analysis. (PDF) [file pmen.0000009.s003.pdf]

Kinyanda *et al. Int J Ment Health Syst (2021) 15:45*
https://doi.org/10.1186/s13033-021-00469-9

International Journal of Mental Health Systems

STUDY PROTOCOL

Open Access

Effectiveness and cost-effectiveness of integrating the management of depression into routine HIV Care in Uganda (the HIV + D trial): A protocol for a cluster-randomised trial

Eugene Kinyanda[1], Leticia Kyohangirwe[1*], Richard S. Mpango[1], Christine Tusiime[1], Joshua Ssebunnya[2], Kenneth Katumba[1], Patrick Tenywa[1], James Mugisha[3], Geoffrey Taasi[4], Hafsa Sentongo[4], Dickens Akena[5], Yoko Laurence[6], Wilson Muhwezi[5], Helen A. Weiss[7], Melissa Neuman[7], Giulia Greco[6], Birthe Knizek[8], Jonathan Levin[9], Pontiano Kaleebu[10], Ricardo Araya[11], Wilber Ssembajjwe[1] and Vikram Patel[12]

Abstract

Background: An estimated 8–30% of people living with HIV (PLWH) have depressive disorders (DD) in sub-Saharan Africa. Of these, the majority are untreated in most of HIV care services. There is evidence from low- and middle-income countries of the effectiveness of both psychological treatments and antidepressant medication for the treatment of DD among PLWH, but no evidence on how these can be integrated into routine HIV care. This protocol describes a cluster-randomised trial to evaluate the effectiveness and cost-effectiveness of the HIV + D model for the integration of a collaborative stepped care intervention for DD into routine HIV care, which we have developed and piloted in Uganda.

Methods: Forty public health care facilities that provide HIV care in Kalungu, Masaka and Wakiso Districts will be randomly selected to participate in the trial. Each facility will recruit 10–30 eligible PLWH with DD and the total sample size will be 1200. The clusters will be randomised 1:1 to receive Enhanced Usual Care alone (EUC, i.e. HIV clinicians trained in Mental Health Gap Action Programme including guidelines on when and where to refer patients for psychiatric care) or EUC plus HIV + D (psychoeducation, Behavioural Activation, antidepressant medication and referral to a supervising mental health worker, delivered in a collaborative care stepwise approach). Eligibility criteria are PLWH attending the clinic, aged ≥ 18 years who screen positive on a depression screening questionnaire (Patient Health Questionnaire, PHQ-9 ≥ 10). The primary outcome is the mean depressive disorder symptom severity scores (assessed using the PHQ-9) at 3 months' post-randomisation, with secondary mental health, disability, HIV and economic outcomes measured at 3 and 12 months. The cost-effectiveness of EUC with HIV + D will be assessed from both the health system and the societal perspectives by collecting health system, patient and productivity costs and mean DD severity scores at 3 months, additional to health and non-health related quality of life measures (EQ-5D-5 L and OxCAP-MH).

*Correspondence: Leticia.Kyohangirwe@mrcuganda.org
[1] MRC/UVRI & LSHTM Uganda Research Unit, Mental Health Section, Entebbe, Uganda
Full list of author information is available at the end of the article

© The Author(s) 2021. This article is licensed under a Creative Commons Attribution 4.0 International License, which permits use, sharing, adaptation, distribution and reproduction in any medium or format, as long as you give appropriate credit to the original author(s) and the source, provide a link to the Creative Commons licence, and indicate if changes were made. The images or other third party material in this article are included in the article's Creative Commons licence, unless indicated otherwise in a credit line to the material. If material is not included in the article's Creative Commons licence and your intended use is not permitted by statutory regulation or exceeds the permitted use, you will need to obtain permission directly from the copyright holder. To view a copy of this licence, visit http://creativecommons.org/licenses/by/4.0/. The Creative Commons Public Domain Dedication waiver (http://creativecommons.org/publicdomain/zero/1.0/) applies to the data made available in this article, unless otherwise stated in a credit line to the data.

Discussion: The study findings will inform policy makers and practitioners on the cost-effectiveness of a stepped care approach to integrate depression management in routine care for PLWH in low-resource settings.

Trial registration: ISRCTN, ISRCTN86760765. Registered 07 September 2017, https://doi.org/10.1186/ISRCTN86760765.

Keywords: Cluster randomised trial, Depression, Routine HIV care, Public health care facilities

Background

An estimated 25 million Africans are living with HIV, and of these, approximately 8–30% have depressive disorders (DD) [1–5]. DD in persons living with HIV (PLWH) not only impair quality of life [6], but are associated with other adverse outcomes such as rapid HIV disease progression including mortality [7, 8], poor adherence to HIV treatment [3, 9, 10], risky sexual behaviour [10, 11] and increased utilisation of health facilities [3, 10]. Despite this, the majority of HIV care providers in sub-Saharan Africa do not routinely provide mental health services [5]. This represents a significant gap as antiretroviral therapy (ART) is scaled-up and HIV transitions into a chronic disease [12]. In contrast, HIV services in some high income countries have successfully integrated mental health care into general HIV care services [13].

The Uganda National HIV and AIDS Strategic Plan (2015–2020) calls for the integration of care for mental disorders and other chronic conditions in HIV care services [14]. To operationalise this recommendation, the Ministry of Health released guidelines for HIV treatment [15] calling for the assessment and management of depression as an integral part of HIV care programs. The aim of the proposed study is to evaluate the effectiveness of the HIV + D intervention on DD, functional outcomes and its cost-effectiveness in routine HIV care in Uganda. The study is led by the Medical Research Council (MRC)/Uganda Virus Research Institute (UVRI) & London School of Hygiene and Tropical Medicine (LSHTM) Uganda Research Unit in partnership with the Ministry of Health.

Currently in Uganda, there is no mental health care in HIV care services provided at public health care facilities (PHCFs) in the country. Challenges to such provision include: (a) low demand for formal mental health services (most patients with mental health problems first seek care from traditional healers and/or faith healers before coming to formal mental health care services); (b) a severe shortage of mental health professionals (Uganda has only about 30 psychiatrists, the majority of whom work in the capital city of Kampala); (c) the reluctance of primary care providers to engage in mental health care (due to lack of training in mental health, appreciation of the value of mental health care and the time and resources due to competing demands) and (d) a severe shortage of primary care workers in most PHCFs (which typically have one clinician for 50–100 patients daily) [16, 17].

Studies from low-and middle-income countries (LMIC) show effectiveness of psychological treatments and antidepressant medication for depressive and anxiety disorders among PLWH in reducing mental health symptoms [18–20] and HIV disease progression [21]. However, a critical knowledge gap remains regarding how such treatments can be integrated in routine HIV care. Integrating improved access to mental health care with HIV care for PLWH has a number of advantages. Firstly, it provides an opportunity to manage two commonly co-occurring disorders to provide a patient-centred approach and remove the barrier of poor communication between providers for the different conditions. Secondly, integration provides us with the opportunity to leverage the limited number of mental health workers as consultants, to improve the capacity of the HIV care delivery platform to address mental illness. Lastly, integrated care programs that address patients' mental health needs in the context of a regular HIV care service are likely to be more attractive to patients and family members who are concerned about the stigma that is still associated with mental illness and their treatment settings [22, 23].

One of the best described and evaluated integration model for mental health is the Collaborative Care (CC) model which provides a pragmatic strategy to deliver integrated mental health and general medical care in primary care settings [24]. CC models provide a team-based, multicomponent intervention that enacts care delivery redesign by systematically improving coordination of patient care through organisational leadership support, evidence-based provider decision-making, and clinical information systems as well as engaging patients in their care through self-management support and linkages to community resources [25]. Systematic reviews have reported that CC models can be a cost-effective strategy for primary care practices to improve mental and physical outcomes for a range of mental health conditions across diverse populations and primary care settings [26, 27]. A systematic review of the constituents of CC interventions which predict favourable outcomes reported the following components: use of routine screening, the professional skills of staff and specialist supervision [28].

There is a growing evidence of the applicability of CC models in LMIC, with several studies showing

effectiveness in management of depression and anxiety disorders in primary care settings [29–31]. In these models, nurses and lay community health workers were employed to provide depression and anxiety management including psychotherapies [29–31]. This task-sharing approach has been shown to encourage effective sharing of tasks between medical, specialist and non-medical staff [32]. Additionally, in the above CC models, mental health treatment was provided in levels or steps ("stepped care") with the most intensive treatments reserved for the most severe cases. CC models that integrate stepped care strategies have been reported to maximise the efficient use of scarce resources, especially in those public health facilities where case management has previously been relatively poor [32].

The depression integration model in the planned trial ("HIV + D") is based on the MANAS intervention [30], a stepped CC delivery model that demonstrated effectiveness for the management of depressive and anxiety disorders delivered by lay health workers in primary health care clinics in India. The HIV + D model is based on therapies that have been shown to be effective against DD in primary care settings including in HIV care, namely: psychoeducation [33], behavioural activation-the Healthy Activity Program (HAP) [34] and antidepressant medications [35, 36].

A similar trial, the CobALT trial is being conducted in HIV care in South Africa [37] to evaluate the effectiveness of mental health integration models in HIV primary care. The HIV + D trial differs from the CobALT trial in that it is being undertaken in Uganda which has a weaker health system than South Africa. In addition, the proposed trial, unlike the CobALT trial, is using an evidence based psychotherapeutic treatment (Behavioural Activation; BA [34].

Developing the HIV + D intervention

We employed robust methodology for intervention development [38] using a systematic, phased approach with the following phases: (I) formative research to inform initial intervention modelling; (II) field testing and refinement in pilot evaluations; (III) a definitive randomised controlled trial.

Phases I and II were undertaken in one peri-urban district of Mpigi in central Uganda from November 2017 to February 2020. These involved: (i) adapting the MANAS collaborative stepped care intervention and a behavioural activation therapy (Health Activity Program-HAP) to the HIV care situation of Uganda, (ii) conducting a pilot study to assess feasibility and acceptability of the developed HIV + D intervention model and (iii) local adaptation and validation of the patient cost questionnaires and the standardised health and non-health quality of life outcome measurement tools (EQ-5D-5 L and OxCAP-MH).

To adapt MANAS, we employed the Theory of Change (ToC) based approach [39], involving consultation with key stakeholders (district health managers and planners, health workers involved in HIV care, representatives of PLWH, Mental health and HIV non-governmental organisations, Ministry of Health representatives from the AIDS Control Programme and the Mental Health Department, and mental health specialists). Three ToC workshops were conducted with stakeholders, resulting in a ToC map that specified the causal pathways, the required interventions, assumptions and indicators to attain the desired impact (improved mental well-being among HIV patients). These outputs were used to inform and further develop the HIV + D intervention.

The local adaptation of behavioural activation therapy (HAP) involved holding consultative meetings with key stakeholders that included mental health experts, HIV clinicians and counsellors and PLWH in order to identify locally appropriate supportive strategies to behavioural activation (BA) that were feasible for delivery by lay health workers (LHW) and acceptable in the HIV care situation of Uganda. The agreed upon supportive strategies to BA were: psychoeducation, problem solving, relaxation, social networks, rumination management and sleep management. The HAP manual and counselor's manual which act as the treatment guide had their text simplified and shortened and translated in the local Luganda language due to low literacy among LHW.

To evaluate the acceptability and feasibility of the HIV + D intervention, a case series of 131 PLWH was undertaken over six months at four public health care facilities in Mpigi district. PLWH were screened and those with a PHQ-9 score ≥ 10 were triaged to the appropriate level of depression management in the HIV + D intervention model. Enrolled patients were followed over 6 months, with monthly assessment of their depressive symptoms using the PHQ-9. In addition, serial qualitative interviews were carried out with clients and lay therapists to gain their perspectives on delivery and use of the intervention. Findings from this formative work were used iteratively to further refine the HIV + D intervention. The developed HIV + D intervention model consists of psychoeducation, Behavioural Activation, antidepressant medication and referral to mental health workers, delivered in a stepped care fashion and on the whole, found to be acceptable to the different stakeholders including PLWH.

After the formative work, the BA sessions were extended from the original 30–40 min to 45–60 min, due to low literacy of both the LHW and study participants; changes were also made to the number of sessions (from

the original 6 optimum sessions to 4 optimum sessions with a range of 4–10 sessions). In addition, the community resources section of the HAP was modified to reflect the key local psychosocial stressors (need for support to orphans and other vulnerable children, domestic violence and land disputes).

The objective of the local adaptation and validation of the health economics instruments was to evaluate the ease of use and understandability of the patient cost questionnaire, EQ-5D-5 L and OxCAP-MH tools which had been translated from English into Luganda. This was achieved through the administration of these tools to a random sample of 20 study respondents drawn from the 4 PHCFs participating in the pilot study. During administration of the tools, participants were encouraged to provide feedback on the Luganda terms used, length of the tools and ease of understanding of the questions. This feedback was used to improve the study tools.

This work showed the feasibility and acceptability of the HIV + D model, with preliminary evidence of a clinically meaningful improvement in DD scores.

Aim and hypothesis

The aim of the planned trial is to evaluate effectiveness of the HIV + D intervention on depressive disorders, functional outcomes and its cost-effectiveness in routine HIV care in Uganda. We hypothesise that, among PLWH with comorbid DD in Uganda, the HIV + D intervention in addition to EUC will be superior to EUC alone in improving clinical and functional DD outcomes, clinical symptoms of generalised anxiety disorder, ART adherence, will be cost-effective and will improve uptake and acceptability of depression management in public HIV care services.

Methods/design

Study setting and design

The study is a cluster-randomised trial with primary endpoint at 3 months' post-randomisation. It will be conducted among 1200 PLWH attending 40 clusters (PHCFs) that provide HIV care. To be eligible, randomly selected PHCFs have to offer HIV care and treatment services to at least 100 PLWH. Eligible clinics will be selected from the 51 PHCFs in the three study districts of Wakiso and Masaka (semi-urban and rural) and Kalungu (rural) which in addition to treating other medical conditions provide HIV care to between 50 and 13,000 PLWH per PHCF depending on size. Randomisation will be completed by an independent statistician using a list of random numbers generated by statistical software (Stata 16.1 SE), and will be stratified by type of health facility (health centres versus hospitals). Within each stratum, clinics will be randomised in a 1:1 ratio to either the Collaborative Stepped Care arm (CSC arm; to receive EUC plus the HIV + D intervention) or the EUC arm (Fig. 1). A minimum of 10 and a maximum of 30 eligible DD positive study participants will be enrolled per participating PHCF. The reporting of the trial will be in accordance with the SPIRIT guidelines for interventional trials [40] (Additional file 1: Appendix 1).

Participant eligibility criteria and recruitment

Inclusion criteria

PLWH who have been on ART for at least 6 months, ≥ 18 years, residing in the study area, medically stable (not too ill to require emergency admission) at the time of the study, conversant in English or Luganda (the predominantly used local language), willing to be home visited (in instances of loss to follow up) and able to give informed consent will be eligible for enrollment if they score ≥ 10 on the PHQ-9.

Exclusion criteria

Potential participants who meet the above inclusion criteria but have impairments that hinder engagement with the research procedures for any reason (e.g. is deaf or hard of hearing, speech impaired, blind or partially sighted), unable to give informed consent, are already receiving treatment for depression or any psychiatric treatment before the trial and those with alcohol use problem will be excluded.

Recruitment

A health talk about depression will be given by the LHW to PLWH sitting in the triage area of the participating PHCFs. Thereafter, consecutive attenders will be approached and requested to be screened for the study until the day's target is reached (4–5 PLWH who have a PHQ-9 score of ≥ 10), screening will be undertaken by the LHW. Selected study respondents will be screened for depression using the PHQ-2, a two-item questionnaire that assess for 'depressed mood' and 'loss of interest in typically pleasurable activities'. Those who screen positive (PHQ-2 score ≥ 3) will be invited for further evaluation by the LHW for trial eligibility and consent. Eligibility assessment will include confirmation of DD using the PHQ-9 [41]. Eligible respondents with suicidal ideation (endorsing item 9 on the PHQ-9) once enrolled will immediately be referred to supervisor for further assessment using MINI to determine suicidality level. Supervisor will consult with or refer to mental health worker for further management plan.

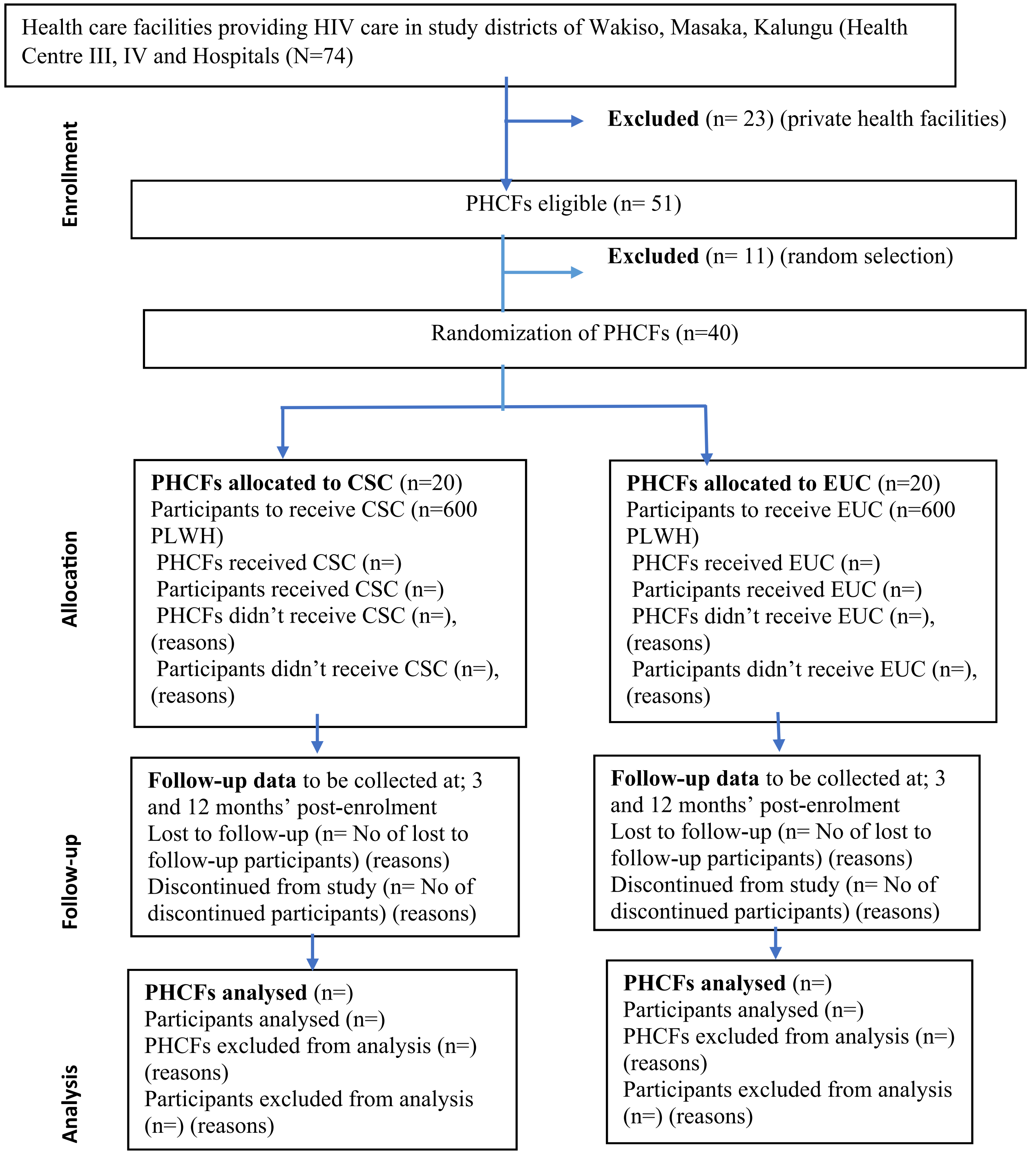

Fig. 1 HIV + D Trial flow-chart: *PHCFs* Public Health Care Facilities, *PLWH* People Living with HIV, *CSC* Collaborative Stepped Care, *EUC* Enhanced Usual Care

Blinding

By design, there is no way to blind the participants on whether or not they receive the intervention. However, the outcome assessors will be blinded to which participants are in the intervention or control group.

Interventions

All participants in the trial will receive Enhanced Usual Care. Usual mental health care in the HIV care situation of Uganda and many other sub-Saharan African settings, is effectively no mental health care. This is primarily because DD is not routinely assessed for or diagnosed. In this study, enhanced usual care will be provided-defined by (i) providing the DD screening results to the attending clinician and (ii) providing mhGAP training to attending clinicians for the target disorders including guidelines on when and where to refer patients for psychiatric care.

In addition to EUC, providers in the intervention arm clinics will receive training in mhGAP and the HIV + D intervention. The HIV + D intervention will consist of evidence-based treatments for depression (psychoeducation, behavioural activation and antidepressant medications) delivered in a stepped care format and overseen by an HIV counsellor/case manager. The HIV + D intervention will be delivered in 4 steps. Step 1: (initiation of treatment), patients with PHQ-9 scores of 10–19 are told of their scores and offered psychoeducation by a LHW. Step 2: (management of moderate to severe cases), patients who remain symptomatic at follow up (PHQ-9 score ≥ 5 after 4 weeks) will be offered BA; 4–10 sessions) by a LHW. Step 3: (monitoring outcomes), if after 6 sessions of BA, PHQ-9 scores are ≥ 10, the advice is to continue BA sessions to completion and add antidepressant medication (Fluoxetine 20 mg/day for 6 months), initiated by the HIV clinician. Step 4: (referral to mental health worker), if there is no improvement (PHQ-9 ≥ 5) after step 3 or at eligibility assessment PHQ-9 ≥ 20, or if someone has a high suicide risk (based on suicide risk assessment using the suicidality module of the Mini International Neuropsychiatric Interview (MINI) [42] by the supervisor for those who score ≥ 1 on item 9 of the PHQ-9 which reads, 'over the last 2 weeks, how often have you had thoughts that you would be better off dead or of hurting yourself in some way?), continue all existing treatment and refer to a mental health worker.

To recruit LHW for this study, a local advert will be run at each of the participating PHCF. Minimum qualifications will include at least 11 years of formal education and a certificate of good conduct from the Local Council I (LC I) leadership, previous training and work in a counsellor role will be an added advantage. Training of LHW will employ both didactic methods and the use of role plays. Supervision of LHWs will be undertaken by the supervising HIV counsellor including through weekly supervision meetings where samples of audio-recordings of the therapy sessions will be listened to and feedback provided.

Fidelity to the intervention

To ensure fidelity in the delivery of the treatment by LHW, the BA therapy sessions have been manualised and all therapy sessions with clients will be audio-recorded. A random sample of these recordings will be discussed with the case manager at weekly clinic meetings. Quantity and quality level fidelity indicators of the treatment will be collected and evaluated. Quantity indicators will be obtained from the study participants' case records where the types of treatment offered, number of sessions per treatment provided and duration of treatment sessions, will be routinely collected by standardised questionnaire. Quality indicators will be assessed through ratings of 10% of the audio-recording of all sessions by independent assessors blind to outcome data using standardised quality assessment questionnaires.

Treatment monitoring and discharge from treatment

The recruited study participants will be followed for 12 months' post-randomisation. The monitoring schedule in the intervention arm will include assessment at 4, 16 and 40 weeks respectively. At these assessment points, the intervention team (LHW and their supervisors) will decide whether the patient should be considered for discharge (PHQ-9 < 5) or stepped up to the next level of care. Patients will be discharged from treatment if their PHQ-9 scores remain below 5 at a follow-up review 4 weeks after a scheduled monitoring visit. Participants with a high suicide risk at any assessment point will be referred to the mental health worker.

Study outcomes measures

The study outcome measures will be collected by means of questionnaires that will be administered to study participants by trained research assistants (TRA) who will be separate from the study implementation team. Data collection by TRA will be supported and overseen by Research Supervisors (RS; trained psychiatric nurses or psychiatric clinical officers who will each oversee four study PHCFs). The three-month outcome is the primary end-point of the trial as majority of study participants will have completed their BA sessions and we would expect the optimal effect of the treatment. The 12-month end-point is included to evaluate the sustainability of the effect of the intervention. The outcome assessment measures with the specific outcomes are summarised in Table 1. The primary outcome measure in this study will be the mean DD symptom severity scores (assessed using the PHQ-9) at 3 months.

Table 1 HIV + D outcome measures and specific outcomes

Outcome	Source of data	Endpoint	
		Collected at 3 months	Collected at 12 months
Primary outcome			
Mean DD symptom severity scores	PHQ-9 [41]	√	
Secondary outcomes			
Mean DD symptom severity scores	PHQ-9 [41]		√
Remission (proportion with PHQ-9 scores < 5)	PHQ-9 [41]	√	√
Mean generalised anxiety disorder (GAD) severity scores	GAD-7 [47]	√	√
Quality adjusted life years (QALYs)	EQ-5D-5 L [48]	√	√
Days out of work (over 3 months)	Patient cost questionnaire	√	√
Functional impairment and welfare	OxCAP-MH [49]	√	√
Patients' satisfaction with depression care	Modified Patient Satisfaction Survey by Ede et al. [50]	√	
Carers' perceptions and satisfaction levels with integration efforts at HIV clinic	Modified Staff Survey by Ede et al. [50]	√	
Proportion with virological failure (a proxy measure of adherence; defined as a viral load of 400 copies/ml or more)	HIV viral load (copies/ml)		√
Reported adherence to ART (proportion of participants who have missed at least one dose of ART in the past three days compared against the baseline value)	Assessed by means of the question, *'Have you missed taking ART in the last 3 days?'* Possible responses 1 = Yes, 2 = No	√	√

Key: Carer = lay health worker, case manager, mental health workers; *ART* antiretroviral therapy

Sample size and power considerations

Power considerations for primary outcome

Twenty clusters will be randomized to each study arm, with the randomisation stratified by type of health facility. The sample size for this trial was calculated using the following assumptions, with testing carried out at the 2.5 % significance level for the primary endpoint to allow for multiple testing:

a. For the mean DD symptom severity score at 3 months.

1. The within-cluster standard deviation in both arms for the DD combined symptom severity score will be at most 4.4 units (based on the results of the INDEPTH trial in Uganda [43], for screen positive subjects).
2. The mean symptom severity score in the CSC arm at 3 months will be 4 units compared to a mean score of 6 units in the EUC arm.
3. The harmonic mean of the number of participants per clinic will be 15.
4. The between cluster coefficient of variation for the PHQ-9 score will be 0.25 in both arms.

Using these assumptions and the formula from Hayes and Moulton [44] (page 110 Eq. 7.9) $C = 1 + (z\alpha/2 + z\beta)2$ $\{2\ \sigma w2\ /\ m + k2\ (\mu 02 + \mu 1\ /\ (\mu 0 - \mu 1)$ [44]; where m is the harmonic mean of the number of participants per clinic, k is the between cluster coefficient of variation, $\sigma w2$ is the within-cluster variance of the DD combined symptom severity scores, $\mu 0$ is the mean DD combined symptom severity score at 3 months in the EUC arm and $\mu 1$ the combined symptom severity score at 3 months in the CSC arm.

Then a sample size of 20 clusters per arm will give 90 % power to detect as statistically significant at the 2.5 % level a true mean difference between the EUC and CSC arms of 2 units in the DD symptom severity score.

The sample size calculation is conservative in having used a large estimate of the within cluster standard deviation, as adjusting for baseline DD score is likely to reduce this.

Data collection and management

Data from study participants at baseline, 3 months, and 12 months' post-enrollment will be collected on printed interviewer-administered face-to-face paper questionnaires which will be stored in lockable filing cabinets at each study site. All the completed questionnaires will be reviewed by a research team member for missing data and unusual responses. Corrective action will be undertaken where necessary by the study research assistant, either by means of a telephone call or by asking the study participant to come back to the clinic. For this, the Data

Section of the MRC/UVRI & LSHTM Uganda Research Unit will design a study database in OpenClinica, using unique study identification-number as a linking field. At regular intervals study questionnaires will be retrieved and transferred to the data section of the MRC/UVRI & LSHTM Uganda Research Unit for double data entry. During data entry, the data manager will periodically carry out a set of pre-defined quality checks on the data and raise any queries with the research assistants through the study coordinator. The OpenClinica database will maintain a complete audit trail of the changes made during data cleaning. Once data for a given survey round in the cluster randomised trial has been cleaned and validated, the data will be locked and exported to Stata for statistical analysis. The dedicated data manager assigned to the study will carry out regular quality control checks on the study database. When all of the queries of a given phase of the study, or a given round of the cluster randomized trial have been addressed, the data manager will upload the cleaned data into the main study database. The main study database will be backed up onto a secure location using a mirror server. In addition, the data manager will maintain a password protected copy of the database on his/her computer and on an external hard drive. A detailed data dictionary will be prepared to help with the curation of the data. The master database for the study will be maintained on the server of the Unit. The data manager and study statisticians will also have password protected copies of the master database.

Statistical analysis

Analyses will be conducted on an intention-to-treat (ITT) basis. For the primary outcome of 'DD symptom severity scores at 3 months' linear mixed models will be fitted to compare the two treatment arms (CSC and EUC) with terms for treatment arm, stratum (level of health facility) and baseline DD symptom severity score, with a random effect fitted for clinic. *A priori* moderators will include gender, social class and baseline severity.

For the binary secondary outcome measures, random effects logistic regression models will be fitted with similar terms to those fitted for the DD severity score analysis. Models with multiple time periods will be estimated using ANCOVA assuming low auto-correlation between time periods [45]. Exploratory longitudinal analyses will be carried out using three-level multilevel models with the levels being clusters, PLWH within clusters and occasions within PLWH (periods). These models will consider (a) the effect of period e.g. whether the symptom score decreases linearly over time and (b) whether there is a treatment arm by period interaction i.e. whether the treatment effect reduces or increases over time.

For the cost-effectiveness component of the study, a micro-costing will be performed to obtain the mean cost per patient, the EQ-5D will be used to construct QALYs and a Markov model will be used to predict the outcomes and cost over the lifetime of the patients [45].

Missing data on outcomes and key covariates will be assessed, and if > 5% of participants are missing data multiple imputation methods appropriate for multilevel data will be used [46].

Monitoring the trial

Four committees will monitor the progress of the trial, that is, Independent Data Monitoring Committee (IDMC), Trial Steering Committee (TSC), Trial Management Team (TMT) and the Trial Management Group (TMG) and details are shown in Additional file 2: Appendix 2.

Ethics and dissemination

The protocol was reviewed and approved by the Uganda Virus Research Institute (UVRI) Research and Ethics Committee (reference number GC/127/20/04/772), the Uganda National Council for Science and Technology (reference number HS645ES), and the London School of Hygiene and Tropical Medicine (LSHTM) Ethics Committee (reference number 22,567). Any protocol modifications will be submitted to the UVRI Research and Ethics Committee for review, and participants will be informed if warranted. The LSHTM is the trial sponsor. Written (or witnessed if the participant is illiterate) informed consent will be mandatory to enrolment. All participant data will be stored securely and access will be restricted. In order to maintain participant confidentiality, all study data collection forms will only be identified by the study identification numbers. Documents that contain participant's names such informed consent forms will be stored separately from data collection forms. All databases will be password protected. HIV-infected persons who do not enrol in the study for any reason will be counselled and referred for care. Serious Adverse Events (SAEs) (death, suicide attempt, hospitalisation) from any cause will be reported as soon as possible within seven calendar days of becoming aware of SAE to the UVRI Research and Ethics Committee and to the IDMC.

Results produced by this investigation will be presented at local and international conferences and published in a timely fashion, ideally in the last year of the study period. All final peer-reviewed manuscripts that arise from this proposal will be submitted to the digital archive PubMed Central for open access.

Discussion

The aim of the HIV + D trial is to provide evidence on the effectiveness and cost-effectiveness of a scalable model for the integration of depression management in HIV care in public health facilities in low resourced settings such as those in Uganda. This intervention model aims to reduce the treatment gap posed by the growing burden of depression among adult persons living with HIV in settings where they currently have no mental health care and where there is an acute shortage of mental health professionals. This project is responding to the Ministry of Health which for the first time in 30 years of the HIV epidemic in Uganda has called for the management of depression in the national HIV prevention and treatment guidelines [15]. These guidelines specifically called for an integrative approach to the management of HIV related comorbidities such as depression [15]. The HIV + D Trial is testing the effectiveness of a health systems intervention based on the MANAS intervention that demonstrated effectiveness for the management of depressive and anxiety disorders in primary care settings in India [30]. A variation of this model, the CobALT trial is currently underway in South Africa [37]. The collaborative care model being evaluated in this trial will be led by lay health care workers under the supervision of HIV care counsellors (general nurses trained in HIV counselling) and supported by mental health workers using a manualised behavioural activation based therapy (the Health Activity Program). The trial design is a cluster randomised trial conducted in 40 HIV care clinics in public health care facilities across three districts in central and south-western Uganda. The study outcomes will be measured on 1200 adult persons living with HIV who have screened positive for depression. It will provide evidence of the effectiveness and cost effectiveness of the intervention on mental, physical and health system outcomes and will highlight the benefits of attending to mental disorders in HIV care programming. Dissemination activities will be organised to share the findings from the cluster randomised trial with stakeholders. An application for further funding will be made to scale up the model if found to be effective.

Abbreviations

ART: Antiretroviral therapy; BA: Behavioural Activation; LHW: Lay health worker; CobALT trial: Acronym for the trial, 'Collaborative care for the detection and management of depression among adults receiving antiretroviral therapy in South Africa'; CSC Arm: Collaborative Stepped Care arm; DD: Depressive Disorders; EQ-5D-5 L: The EuroQol 5 dimensions; EUC Arm: Enhanced Usual Care arm; HIV + D: Acronym for the project, 'Integrating the management of depression into routine HIV care in Uganda'; HIV + D Model: HIV + D Interventional model; LMIC: Low and middle income countries; MANAS: Acronym for the cluster randomised trial that was implemented in India to evaluate a stepped care collaborative delivery model for depression and anxiety disorders in primary health care; mhGAP: WHO's Mental Health Gap Action Programme; WHO: World Health Organisation; MOH: Ministry of Health; MRC/UVRI & LSHTM Uganda Research Unit: Medical Research Council / Uganda Virus Research Unit & London School of Hygiene and Tropical Medicine Uganda Research Unit; OxCAP-MH: Oxford Capabilities Questionnaire for Mental Health; PHCF: Public health care facilities; PHQ-9: The Patient Health Questionnaire-9; PLWH: People Living with HIV; ToC: Theory of Change; UNCST: Uganda National Council for Science and Technology; UVRI: Uganda Virus Research Institute; REC: Research Ethics Committee; HIV/AIDS: Human immunodeficiency virus and acquired immune deficiency syndrome.

Supplementary Information

The online version contains supplementary material available at https://doi.org/10.1186/s13033-021-00469-9.

Additional file 1: Appendix 1. SPIRIT 2013 checklist. **Additional file 2: Appendix 2.** Trial management committee.

Acknowledgements

Patients and staff of the HIV clinics at Mpigi Health Centre IV, Nkozi Hospital, Buwama Health Centre III and Butooro Health Centre III in Mpigi district who participated in the formative research of this project.

Authors' contributions

Writing first draft and reviewing subsequent drafts leading to the final manuscript: LK, EK; review of first draft and subsequent drafts: VP, MN, HAW, YL, RSM, CT, JS, KK, PT, JM, JL; review of final draft: VP, RA, GT, GG, BK, WS, HS, DA, WM, JL, PK. All authors read and approved the final manuscript.

Funding

This cluster randomised trial is funded by Wellcome Trust. This has no role in the design of the study and collection, analysis, and interpretation of data and in writing the manuscript. The trial is sponsored by the London School of Hygiene and Tropical Medicine and have no role in the design of the study and collection, analysis, and interpretation of data and in writing the manuscript.

Availability of data and materials

Data sharing is not applicable to this protocol as no datasets are generated or analysed yet.

Declarations

Ethics approval and consent to participate

The protocol was reviewed and approved by the Uganda Virus Research Institute (UVRI) Research and Ethics Committee (reference number GC/127/20/04/772), the Uganda National Council for Science and Technology (reference number HS645ES), and the London School of Hygiene and Tropical Medicine (LSHTM) Ethics Committee (reference number 22567). Informed consent will be obtained from each potential participant before enrolment into the study.

Consent for publication

Not applicable.

Competing interests

The authors declare that they have no competing interests.

Author details

[1]MRC/UVRI & LSHTM Uganda Research Unit, Mental Health Section, Entebbe, Uganda. [2]Butabika National Referral Mental Hospital, Kampala, Uganda. [3]Kyambogo University, Kampala, Uganda. [4]Ministry of Health, Kampala, Uganda. [5]Department of Psychiatry, Makerere University College of Health Sciences, Kampala, Uganda. [6]Department for Global Health and Development, Public Health and Policy, London School of Hygiene & Tropical Medicine, Centre for Health Economics in London, London, England. [7]MRC International Statistics and Epidemiology Group LSHTM, London, England. [8]Department of Psychology, Norwegian University of Science and Technology, Trondheim,

Norway. [9]Department Statistics, University of the Witwatersrand, Johannesburg, South Africa. [10]MRC/UVRI & LSHTM Uganda Research Unit, Entebbe, Uganda. [11]Centre for Global Mental Health, Kings College London, London, England. [12]Department of Global Health and Social Medicine, Harvard Medical School, Boston, Massachusetts, USA.

Received: 4 January 2021 Accepted: 2 May 2021
Published online: 12 May 2021

References

1. UNAIDS. (2019) Global HIV and AIDS statistics. https://www.unaids.org/en/resources/fact-sheet [cited 24 June 2020].
2. Kinyanda E, Hoskins S, Nakku J, Nawaz S, Patel V. Prevalence and risk factors of major depressive disorder in HIV/AIDS as seen in semi-urban Entebbe district, Uganda. BMC Psychiatry. 2011;11(1):205.
3. Kinyanda E, Nakasujja N, Levin J, Birabwa H, Mpango R, Grosskurth H, et al. Major depressive disorder and suicidality in early HIV infection and its association with risk factors and negative outcomes as seen in semi-urban and rural Uganda. J Affect Disord. 2017;212:117–27.
4. Myer L, Smit J, Roux LL, Parker S, Stein DJ, Seedat S. Common mental disorders among HIV-infected individuals in South Africa: prevalence, predictors, and validation of brief psychiatric rating scales. AIDS Patient Care STDs. 2008;22(2):147–58.
5. Abas M, Ali GC, Nakimuli-Mpungu E, Chibanda D. Depression in people living with HIV in sub-Saharan Africa: time to act. Trop Med Int Health. 2014;19(12):1392–6.
6. Douaihy A, Singh N. Factors affecting quality of life in patients with HIV infection. AIDS Read. 2001;11(9):450–4. 60 – 1, 75.
7. Farinpour R, Miller EN, Satz P, Selnes OA, Cohen BA, Becker JT, et al. Psychosocial risk factors of HIV morbidity and mortality: findings from the Multicenter AIDS Cohort Study (MACS). J Clin Exp Neuropsychol. 2003;25(5):654–70.
8. Langford SE, Ananworanich J, Cooper DA. Predictors of disease progression in HIV infection: A review. AIDS Res Ther. 2007;4(1):11.
9. Springer SA, Dushaj A, Azar MM. The impact of DSM-IV mental disorders on adherence to combination antiretroviral therapy among adult persons living with HIV/AIDS: a systematic review. AIDS Behav. 2012;16(8):2119–43.
10. Kinyanda E, Levin J, Nakasujja N, Birabwa H, Nakku J, Mpango R, et al. Major depressive disorder: Longitudinal analysis of impact on clinical and behavioral outcomes in Uganda. J Acquir Immune Defic Syndr. 2018;78(2):136–43.
11. Seth P, Patel SN, Sales JM, DiClemente RJ, Wingood GM, Rose ES. The impact of depressive symptomatology on risky sexual behavior and sexual communication among African American female adolescents. Psychol Health Med. 2011;16(3):346–56.
12. Colvin CJ. HIV/AIDS, chronic diseases and globalisation. Globalization Health. 2011;7(1):31.
13. Collins PY, Holman AR, Freeman MC, Patel V. What is the relevance of mental health to HIV/AIDS care and treatment programs in developing countries? A systematic review. AIDS. 2006;20(12):1571.
14. Uganda AIDSC: The Uganda National HIV and Strategic Plan AIDS. 2015/2016–2019/2020. 2015, http://library.health.go.ug/publications/service-delivery-diseases-control-prevention-communicable-diseases/hivaids/national-h-1.
15. Ministry of Health, Uganda. Consolidated guidelines for the prevention and treatment of HIV in Uganda. December 2016. MOH, Uganda.
16. Wagner GJ, Ngo V, Glick P, Obuku EA, Musisi S, Akena D. INtegration of DEPression Treatment into HIV Care in Uganda (INDEPTH-Uganda): study protocol for a randomized controlled trial. Trials. 2014;15(1):248.
17. Kigozi FN, Ssebunnya J. Integration of mental health into primary health care in Uganda: opportunities and challenges. Mental Health Family Med. 2009;6(1):37.
18. Li L, Lee S-J, Jiraphongsa C, Khumtong S, Iamsirithaworn S, Thammawijaya P, et al. Improving the health and mental health of people living with HIV/AIDS: 12-month assessment of a behavioral intervention in Thailand. Am J Public Health. 2010;100(12):2418–25.
19. Nakimuli-Mpungu E, Wamala K, Okello J, Alderman S, Odokonyero R, Musisi S, et al. Outcomes, feasibility and acceptability of a group support psychotherapeutic intervention for depressed HIV affected Ugandan adults: A pilot study. J Affect Disord. 2014;166:144–50.
20. Onu C, Ongeri L, Bukusi E, Cohen CR, Neylan TC, Oyaro P, et al. Interpersonal psychotherapy for depression and posttraumatic stress disorder among HIV-positive women in Kisumu, Kenya: study protocol for a randomized controlled trial. Trials. 2016;17(1):64.
21. Nyaga I: Efficacy and acceptability of Interpersonal Therapy on depressed HIV/AIDS people attending city county health facilities in Dagorretti District, Nairobi. A PhD thesis, Nairobi University, 2016.
22. Freeman MC, Patel V, Collins PY, Bertolote JM. Integrating mental health in global initiatives for HIV/AIDS. The British Journal of Psychiatry. 2005;187(1):1–3.
23. Patel V, Belkin GS, Chockalingam A, Cooper J, Saxena S, Unützer J. Grand challenges: integrating mental health services into priority health care platforms. PloS medicine. 2013;10(5):e1001448.
24. Unützer J, Park M. Strategies to improve the management of depression in primary care. Prim Care Clinics Office Pract. 2012;39(2):415–31.
25. Goodrich DE, Kilbourne AM, Nord KM, Bauer MS. Mental health collaborative care and its role in primary care settings. Current Psychiatr Rep. 2013;15(8):383.
26. Jacob V, Chattopadhyay SK, Sipe TA, Thota AB, Byard GJ, Chapman DP, et al. Economics of collaborative care for management of depressive disorders: a community guide systematic review. Am J Prev Med. 2012;42(5):539–49.
27. Woltmann E, Grogan-Kaylor A, Perron B, Georges H, Kilbourne AM, Bauer MS. Comparative effectiveness of collaborative chronic care models for mental health conditions across primary, specialty, and behavioral health care settings: systematic review and meta-analysis. Am J Psychiatry. 2012;169(8):790–804.
28. Bower P, Gilbody S, Richards D, Fletcher J, Sutton A. Collaborative care for depression in primary care: making sense of a complex intervention: systematic review and meta-regression. Br J Psychiatr. 2006;189(6):484–93.
29. Araya R, Flynn T, Rojas G, Fritsch R, Simon G. Cost-effectiveness of a primary care treatment program for depression in low-income women in Santiago, Chile. Am J Psychiatr. 2006;163(8):1379–87.
30. Patel V, Weiss HA, Chowdhary N, Naik S, Pednekar S, Chatterjee S, et al. Effectiveness of an intervention led by lay health counsellors for depressive and anxiety disorders in primary care in Goa, India (MANAS): a cluster randomised controlled trial. Lancet. 2010;376(9758):2086–95.
31. Chibanda D, Mesu P, Kajawu L, Cowan F, Araya R, Abas MA. Problem-solving therapy for depression and common mental disorders in Zimbabwe: piloting a task-shifting primary mental health care intervention in a population with a high prevalence of people living with HIV. BMC Public Health. 2011;11(1):828.
32. Buttorff C, Hock RS, Weiss HA, Naik S, Araya R, Kirkwood BR, et al. Economic evaluation of a task-shifting intervention for common mental disorders in India. Bull World Health Organ. 2012;90:813–21.
33. Ali BS, Rahbar MH, Naeem S, Gul A, Mubeen S, Iqbal A. The effectiveness of counseling on anxiety and depression by minimally trained counselors: a randomized controlled trial. Am J Psychother. 2003;57(3):324–36.
34. Chowdhary N, Anand A, Dimidjian S, Shinde S, Weobong B, Balaji M, et al. The Healthy Activity Program lay counsellor delivered treatment for severe depression in India: systematic development and randomised evaluation. Br J Psychiatr. 2016;208(4):381–8.
35. Olatunji BO, Mimiaga MJ, O'Cleirigh C, Safren SA. Review of treatment studies of depression in HIV. Topics in HIV medicine: a publication of the International AIDS Society, USA. 2006;14(3):112 – 24.
36. Rabkin JG, Wagner GJ, Rabkin R. Fluoxetine treatment for depression in patients with HIV and AIDS: a randomized, placebo-controlled trial. Am J Psychiatry. 1999;156(1):101–7.
37. Fairall L, Petersen I, Zani B, Folb N, Georgeu-Pepper D, Selohilwe O, et al. Collaborative care for the detection and management of depression among adults receiving antiretroviral therapy in South Africa: study protocol for the CobALT randomised controlled trial. Trials. 2018;19(1):193.
38. Patel V, Weobong B, Weiss HA, Anand A, Bhat B, Katti B, et al. The Healthy Activity Program (HAP), a lay counsellor-delivered brief psychological treatment for severe depression, in primary care in India: a randomised controlled trial. Lancet. 2017;389(10065):176–85.
39. De Silva MJ, Breuer E, Lee L, Asher L, Chowdhary N, Lund C, et al. Theory of Change: a theory-driven approach to enhance the Medical Research Council's framework for complex interventions. Trials. 2014;15:267.

40. Chan AW, Tetzlaff JM, Altman DG, Laupacis A, Gøtzsche PC, Krleža-Jerić K, et al. SPIRIT 2013 statement: defining standard protocol items for clinical trials. Ann Intern Med. 2013;158(3):200–7.
41. Kroenke K, Spitzer RL. The PHQ-9: a new depression diagnostic and severity measure. Psychiatric Annals. 2002;32(9):509–15.
42. Sheehan DV, Lecrubier Y, Sheehan KH, Amorim P, Janavs J, Weiller E, et al. The Mini-International Neuropsychiatric Interview (M.I.N.I.): the development and validation of a structured diagnostic psychiatric interview for DSM-IV and ICD-10. J Clin Psychiatry. 1998;59 Suppl 20:22–33;quiz 4–57.
43. Wagner GJ, Ngo V, Goutam P, Glick P, Musisi S, Akena D. A structured protocol model of depression care versus clinical acumen: a cluster randomized trial of the effects on depression screening, diagnostic evaluation, and treatment uptake in Ugandan HIV clinics. PloS ONE. 2016;11(5):e0153132.
44. Hayes R, Moulton L. Introduction. Cluster Randomised Trials, Interdisciplinary Statistics. Boca Raton: Chapman and Hall/CRC; 2009.
45. Drummond MF, Sculpher MJ, Claxton K, Stoddart GL, Torrance GW. Methods for the economic evaluation of health care programmes: Oxford university press; 2015.
46. Drechsler J. Multiple imputation of multilevel missing data—Rigor versus simplicity. J Educ Behav Statistics. 2015;40(1):69–95.
47. Spitzer RL, Kroenke K, Williams JB, Löwe B. A brief measure for assessing generalized anxiety disorder: the GAD-7. Arch Intern Med. 2006;166(10):1092–7.
48. Group TE. EuroQol-a new facility for the measurement of health-related quality of life. Health Policy. 1990;16(3):199–208.
49. Vergunst F, Jenkinson C, Burns T, Anand P, Gray A, Rugkåsa J, et al. Psychometric validation of a multi-dimensional capability instrument for outcome measurement in mental health research (OxCAP-MH). Health Qual Life Outcomes. 2017;15(1):1–11.
50. Ede V, Okafor M, Kinuthia R, Belay Z, Tewolde T, Alema-Mensah E, et al. An examination of perceptions in integrated care practice. Commun Ment Health J. 2015;51(8):949–61.

Publisher's Note

Springer Nature remains neutral with regard to jurisdictional claims in published maps and institutional affiliations.

Ready to submit your research? Choose BMC and benefit from:

- fast, convenient online submission
- thorough peer review by experienced researchers in your field
- rapid publication on acceptance
- support for research data, including large and complex data types
- gold Open Access which fosters wider collaboration and increased citations
- maximum visibility for your research: over 100M website views per year

At BMC, research is always in progress.

Learn more biomedcentral.com/submissions

BMC
