# Supplementary material for: Process evaluation of the HIV+D intervention for integrating the management of depression in routine HIV care in Uganda
Source: PLOS Ment Health. 2024 Jun 4;1(1):e0000009. doi: 10.1371/journal.pmen.0000009 (PMC12798554; doi:10.1371/journal.pmen.0000009)
Supplement: Attachment — Submitted filename: Dear Editor.docx [file pmen.0000009.s004.docx]

Dear Editor,

Thank you for checking the submission, and raising the issues, for improvement of the manuscript.

I have uploaded other copies of the ethics approval letters, covering the date range for participant recruitment and beyond.

The manuscript has also been revised to address the issue of significant text overlap between the current submission and some other previously published work; rephrasing the duplicated texts.

Once again, thank you for raising these concerns.

Warm regards,

Joshua
