# Supplementary material for: Process evaluation of the HIV+D intervention for integrating the management of depression in routine HIV care in Uganda
Source: PLOS Ment Health. 2024 Jun 4;1(1):e0000009. doi: 10.1371/journal.pmen.0000009 (PMC12798554; doi:10.1371/journal.pmen.0000009)
Supplement: Attachment — Submitted filename: Response to Reviewers.docx [file pmen.0000009.s005.docx]

Dear Editor,

We thank you and the reviewers for the useful comments. We have revised the manuscript, consequently addressing the concerns raised, as indicated below in the mail and highlighted in the revised manuscript.

1. Please provide separate figure files in .tif or .eps format.

The latest comment from the editor instructed that the table shouldn’t be provided as a separate file in .tif. Instead, it should be included in the manuscript file as editable, cell-based objects. This is what has been done.

Please review your reference list to ensure that it is complete and correct. If you have cited papers that have been retracted, please include the rationale for doing so in the manuscript text, or remove these references and replace them with relevant current references. Any changes to the reference list should be mentioned in the rebuttal letter that accompanies your revised manuscript. If you need to cite a retracted article, indicate the article’s retracted status in the References list and also include a citation and full reference for the retraction notice.

The reference list has been revised, replacing most of the references above 5 years, with the more recent ones; save for a few that just couldn’t be substituted. Also there are no retracted papers that are cited in the revised reference list.

Additional Editor Comments (if provided):

In addition to the comments raised by the reviewers, kindly address these comments from the editor.

• Too many abbreviations without prior definition e.g., MRC, mhGAP

The abbreviations have been written in full on first mention

• How was the participating sites selected? Any selection criteria?

We have revised the section, highlighting how the participating sites were selected.

• I doubt if “Reach” can easily be assessed in this design. I think “Reach” should be the number of persons with HIV who received screening for depression or those who received the intervention for depression. I believe, the authors can easily assess acceptability, fidelity, sustainment and the context.

We appreciate the correction, and have revised this section accordingly, indicating that this process evaluation assessed the other aspects other than “Reach”.

• The acceptability was reported only at the client/patient level. What about acceptability at the level of health care providers and the participating institutions?

We have made the revision, reporting on acceptability at the level of health care providers as well.

• The acceptability dimension also measures satisfaction to intervention as mentioned by the authors. In addition to the qualitative method, addition of a validated patient satisfactiom questionnaire would have made it more rigorous.

Indeed, a validated patient satisfaction questionnaire would have made it more rigorous. However, we didn’t administer one, as it was a purely qualitative study. We have reported on patient satisfaction as reflected in the qualitative interviews. However, we acknowledge this as one of the limitations.

• In the context of implementation, the authors highlighted the barriers to to successful intervention but were silent about the qualities of the environment or the intervention that facilitated the success of the program.

We have revised this section slightly, reporting on the qualities of the intervention and other contextual factors that facilitated success. However, we are silent about the environmental factors in the findings section because the respondents made no mention of the environmental or topographical factors that facilitated the success of the program.

• What the limitations of this study?

We have added a section on the limitations of the study.

Reviewers' comments:

5. Review Comments to the Author

Reviewer #1: I would suggest 2 minor changes to the article: 1) it would be great to have more extended conclusions, so they represent all the major findings, not just the policy recommendations, 2) the data that can be made available, must be made available, there are formats for the qualitative data availability; please consider this. Otherwise the article is great :-)

This has been addressed. The conclusion has been revised accordingly.

Also the data (transcriptions) are available and will be sent to the editor when instructed.

Reviewer #2: Method:

1. what is DD? please write in full before using the acronym.

This has been addressed.

2. Fluoxetine 20mg/day/6months: Did every patient on this regimen respond and tolerate the medication for the 6/12?

According to the reports at the facilities, the few patients on the regimen responded positively, as there were no complaints or further referrals

3. Who precisely do you refer to as Mental Health Worker? Please, specify.

We have revised this to mental health specialist, specifying who is referred to as mental health specialist.

Result:

1. in table 1, No. of interviewees (not 'interviews') you mean?

This has been revised to ‘No. of Key Informant Interviews’ (as the row that follows is for No. of FGDs)

Ethical consideration:

1. state the date the ethical approval was issued.

The dates for ethical approval by the different bodies have been stated, as advised.

Reference:

Majority of your references cited were old (more than 5 years of publication). You may update them with newer studies as much as possible.

The reference list has been updated with newer studies.

Most cordial regards,

Joshua
